# Polish Medical Air Rescue Crew Interventions Concerning Neonatal Patients

**DOI:** 10.3390/children8070557

**Published:** 2021-06-29

**Authors:** Ewa Rzońca, Grażyna Bączek, Marcin Podgórski, Robert Gałązkowski

**Affiliations:** 1 Department of Education and Research in Health Sciences, Faculty of Health Sciences, Medical University of Warsaw, 02-091 Warsaw, Poland; 2 Department of Obstetrics and Gynecology Didactics, Faculty of Health Sciences, Medical University of Warsaw, 00-575 Warsaw, Poland; grazyna.baczek@wum.edu.pl; 3 Department of Emergency Medical Services, Faculty of Health Sciences, Medical University of Warsaw, 02-091 Warsaw, Poland; marcin.podgorski@wum.edu.pl (M.P.); r.galazkowski@lpr.com.pl (R.G.)

**Keywords:** emergency medical services, newborn, patients, air ambulance

## Abstract

The purpose of the study was to present the characteristics of Helicopter Emergency Medical Service (HEMS) and Emergency Medical Service (EMS) interventions concerning newborns in Poland. The study involved a retrospective analysis of missions by Polish Medical Air Rescue crews concerning newborns, carried out in Poland between January 2011 and December 2020. Polish Medical Air Rescue crews were most commonly dispatched to urban areas (86.83%), for patient transfer (59.67%), using an airplane (65.43%), between 7 AM and 6:59 PM (93.14%), and in the summer (28.67%). Further management involved handing over the neonatal patient to a ground neonatal ambulance team. Most of the patients studied were male (58.02%), and the most common diagnosis requiring the HEMS or EMS intervention was a congenital heart defect (31.41%). The most common medical emergency procedure performed by Polish Medical Air Rescue crew members for the neonatal patients was intravenous cannulation (43.07%). The odds ratio for congenital malformations was higher in male newborns. The type of Polish Medical Air Rescue mission was associated with the location of the call, time of the call, ICD-10 diagnosis associated with the dispatch, selected clinical findings, most commonly performed medical emergency procedures, and mission duration and distance covered.

## 1. Introduction

The task of Helicopter Emergency Medical Service (HEMS) teams, similar to ground emergency medical teams, is to respond to emergencies and perform emergency procedures both on scene and during transportation of the patient to the hospital [1,2]. A HEMS crew consists of a professional pilot, and personnel authorized to perform emergency medical procedures, i.e., a physician and an emergency medical technician or nurse. In Poland, air medical rescue teams are dispatched by the Polish Medical Air Rescue, which operates both HEMS teams and an airplane-based Emergency Medical Service (EMS) transport team. The EMS crew is composed of two professional pilots, a physician, and an emergency medical technician or nurse. Currently, 21 Polish Medical Air Rescue bases exist in Poland, including four 24 h bases and one seasonal base operating in the summer [2]. Polish Medical Air Rescue missions are carried out using two types of aircraft: the Piaggio P.180 Avanti airplane (cruise speed—745 km/h, range—2722 km) and EC 135 helicopters (cruise speed—254 km/h, range—608 km) [3]. The HEMS crew readiness for take-off depends on the time of day or night and distance to the scene. During the day, HEMS crews are ready for flights up to 60 km within 3 min, 60–130 km—within 6 min, and above 130 km—within 15 min. During the night, this time is up to 15 min for flights not exceeding 60 km, and up to 30 min for longer flights [2,4]. In the case of EMS crews, both during the day and night, the time is up to 60 min for the first call of the day, and up to 30 min for any subsequent call on the same day [4].

The purpose of the HEMS, as one of the pillars of the Polish medical emergency response system, is to provide pre-hospital care to patients of any age with severe or life-threatening emergencies [2]. Pediatric patients are of particular interest to researchers in the context of pre-hospital care and transport to the hospital [5,6,7,8]. One special group of pediatric patients includes newborns [9,10,11]. It is worth noting that modern advances in terms of new medical technologies and the longer life expectancy of premature infants are contributing to the growing demand for increasingly specialized care [10,11]. This entails the need to organize and provide multi-dimensional perinatal care to the pregnant woman, the fetus, and the newborn. Neonatal transport is performed with a view to providing quality, individualized medical care to newborns in a healthcare facility with an appropriate referral level [12,13,14]. Polish Medical Air Rescue HEMS crews do not only perform inter-center patient transfers and provide care for their duration—they are also used for emergency response when the incident location is inaccessible to ground units due to challenging topography or when the time of arrival on scene or the duration of transport to the hospital is a key factor in patient risk [2].

The importance of air medical rescue for pre-hospital care, combined with the unique characteristics of the neonatal patient group, motivated the present study. Its purpose was to present the characteristics of HEMS and EMS interventions concerning newborns in Poland.

## 2. Materials and Methods

The study involved a retrospective analysis of missions by HEMS and EMS crews of the Polish Medical Air Rescue concerning newborns in Poland. The analysis included all HEMS and EMS flights to emergency cases, as well as inter-center patient transfer missions, where medical assistance had been provided to newborns, in Poland between January 2011 and December 2020. Cases where the transport order had been canceled or the mission had not been performed, due to weather conditions or technical problems, were excluded from analysis. Ultimately, based on the established criteria, 729 cases of missions concerning newborns completed by HEMS and EMS crews were included in the analysis. The study was approved by the director of the Polish Medical Air Rescue. As this was a retrospective study, it did not require consent, as confirmed by the Bioethics Committee of the Medical University of Warsaw in Poland, which had received the study protocol (AKBE/81/2021).

The study used medical and operational records of HEMS and EMS crews of the Polish Medical Air Rescue. We performed an analysis of Polish Medical Air Rescue crews’ electronic records comprising 89,726 missions carried out by HEMS and EMS crews. Based on the study criteria, we identified 729 cases, which were then further verified and complemented based on information from hard-copy medical and operational records of the Polish Medical Air Rescue. The documentation was analyzed to obtain the following information: patient sex, mission date, main diagnosis in accordance with the International Statistical Classification of Diseases and Related Health Problems (ICD-10), emergency procedures performed, clinical parameters of the patients, and other intervention characteristics.

STATISTICA software, version 13.2 (Tibco Software Inc., Palo Alto, CA, United States), was used to analyze the data obtained. Numbers (*n*) and percentages (%) were used to describe the qualitative data, whereas means (M), standard deviations (SD) and interquartile range (IQR) were used to report the quantitative data. The Lilliefors test and the Kolmogorov–Smirnov test were used to determine the normality of distribution for the quantitative variables. The chi-squared statistic was used to test statistically significant differences between the qualitative variables, and the nonparametric Mann–Whitney U-test was used to compare differences between two independent groups. To determine the odds of specific health problems occurring in the newborns depending on their sex, we used odds ratios (ORs) and 95% confidence intervals (95% CI). The significance level adopted in the study was *p* < 0.05.

## 3. Results

Polish Medical Air Rescue crews were most commonly dispatched to urban areas (86.83%), for patient transfer (59.67%), using an airplane (65.43%), between 7 AM and 6:59 PM (93.14%), and in the summer (28.67%). Further management involved handing over the neonatal patient to a ground neonatal ambulance team (64.06%). The mean total duration of the mission was 244.37 min. The mean distance to the scene was 251.12 km, and the mean distance to the hospital was 250.30 km. Detailed data are shown in Table 1.

Most of the newborns studied were male (58.02%), and the most common diagnosis requiring the HEMS or EMS intervention was a congenital heart defect (31.41%). The most common medical emergency procedures performed by Polish Medical Air Rescue crew members for the newborn patients were intravenous cannulation (43.07%) and mechanical ventilation (22.36%). Details on the characteristics of the newborns, the most commonly performed emergency medical procedures, and test results are shown in Table 2.

Emergency missions were more frequent in rural areas (60.81%), and the most common diagnoses requiring this type of Polish Medical Air Rescue crew intervention included respiratory failure (35.14%), sudden cardiac arrest (18.92%), and other health problems (41.22%). With this mission type, as opposed to rescue transport or patient transfer, most common clinical findings in the newborns studied were apnea (17.57%) and cyanosis (31.08%), and the most often performed procedure was oxygen therapy (31.08%). Moreover, during flights to emergencies, the duration of the flight to the scene, transport to the hospital, and patient care were considerably shorter than in other types of missions, as were the distances covered. These findings were statistically significant at *p* < 0.05. On the other hand, there was no statistically significant association between the type of mission and the clinical findings of dyspnea in the neonatal patients (*p* > 0.05). Detailed data are shown in Table 3.

Female newborns had higher odds ratios for respiratory failure, sudden cardiac arrest, and other health problems—Table 4.

## 4. Discussion

The neonatal period is among the most exceptional and critical parts of human life, requiring care adjusted to the needs and health of the newborn baby [15]. The study by Giri et al. demonstrated that newborns were the third largest patient group requiring air transport in the first year of Helicopter Emergency Medical Services operation in Bhutan. In addition, the authors found that the use of a specialized helicopter crew for care and transport was associated with better outcomes, and especially a higher survival rate in the newborns [16]. Romanzeira and Sarinho (2015) reported that most newborns transported by the Mobile Emergency Medical Services in Recife, Brazil, were male and required transport due to respiratory failure [10]. Akula et al. (2016) reported on neonatal transport cases in California, USA. They found that the transported infants were more likely to be male, and that nearly one third were diagnosed with congenital malformations [9]. In our study, care and transport by HEMS and EMS crews in Poland were required more often in cases of male newborns, with the most common diagnoses involving congenital malformations, and especially congenital heart defects. Most missions involved patient transfer by airplane, called in from urban areas. The odds ratio for these conditions was also higher among male newborns. Xie et al. (2016) likewise found higher odds ratios for congenital malformations in male newborns and in those from urban areas, and a similar observation on the role of sex in this context was reported by Zhou et al. (2020) [17,18].

According to Akula et al. (2016), helicopters were most often used for emergency transport, compared to other modes of transport and mission types [9]. In turn, our study demonstrated that emergency response was more often provided by Polish Medical Air Rescue teams in cases where the diagnosis involved respiratory failure, sudden cardiac arrest, and other health problems, while rescue transport and patient transfer flights were carried out most often in cases of newborns with congenital malformations. Kempley et al. (2004) studied the number and characteristics of inter-hospital neonatal transfers in London and south-east England. They reported that, on average, the daily number of elective transfers was highest, followed by urgent transfers and the least numerous short-term transfers [19].

It should be noted that stabilizing the condition of newborns before transport helps reduce the adverse event risk. This is important, as such events may be the cause of mortality or morbidity. Neonatal transport services allow for providing intensive care to newborns in critical condition before and during transfer between healthcare facilities, due to the performance of the required emergency medical procedures, among other factors [20,21,22,23]. The study by Kumar et al. (2011) demonstrated that the emergency procedures most frequently carried out during neonatal transport by specialist team members involved intravenous cannulation and fluid resuscitation [22]. Wahab et al. (2019) analyzed factors affecting the duration of infant stabilization before transport. They reported that the most commonly performed medical procedures included intravenous cannulation, and intubation and ventilation [23], which is also confirmed by our present findings. In addition, Wahab et al. (2019) found that in cases of newborns requiring one or more medical procedures or interventions, the stabilization took a significantly longer time than in cases of transfers without interventions [23]. Our present findings indicate that Polish Medical Air Rescue teams performed the most emergency medical procedures during medical rescue transport missions, and the time spent on scene and the patient care duration were longest with this type of mission. According to Akula et al. (2016), the median patient transport duration was 2.2 h [9], while in Romanzeira and Sarinho (2015), the mean neonatal transport duration was 58 min [10]. Notably, the priorities of emergency medical services include rapid response, immediate initiation of life-saving emergency medical procedures on scene, and rapid patient transport to a health care facility capable of providing the medical care required. This is why time indicators, such as time to call (i.e. time between the incident and notification of the relevant responders), response time (time between receipt of the call by an emergency medical team and take-off from their stand-by location), time spent on scene (time between the arrival of the team on scene and subsequent take-off—beginning of transport), and transport time (time between take-off from the scene and arrival at the target health care facility), are of utmost importance in the work of medical emergency units [24,25,26].

According to our knowledge, the present study is the first 10 year retrospective analysis of Polish Medical Air Rescue’s HEMS and EMS missions involving neonatal patients in Poland. In addition, the present study complements our previous study concerning the transport of patients in incubators [27]. We acknowledge that our study had a number of limitations that we are aware of, which may, however, provide some guidance for further investigations on the subject. The information included in the analysis was derived from the Polish Medical Air Rescue HEMS and EMS crews’ documentation, with no data on the newborns’ maturity at birth or outcomes after patient handover. These limitations do not, however, impair the quality of the study. Further research on neonatal care and transport provided by HEMS and EMS crews should result in an even better understanding of the issues being analyzed, so that the best possible quality of care can be ensured for neonatal patients.

## 5. Conclusions

Polish Medical Air Rescue crews were most commonly dispatched to newborns in rural areas, for patient transfer by airplane, and during the day and in the summer, and the patients were then most commonly handed over to a ground neonatal ambulance team.

Polish Medical Air Rescue teams were most commonly dispatched to cases of male newborns, and the underlying diagnoses most often involved congenital malformations, and especially congenital heart defects. The odds ratio for congenital malformations was higher in male newborns.

The type of Polish Medical Air Rescue missions was associated with the location of the call, time of the call, ICD-10 diagnosis associated with the dispatch, clinical findings (except for dyspnea), most commonly performed medical emergency procedures, and mission duration and distance covered (specific mission details).

## Figures and Tables

**Table 1:** Characteristics of Polish Medical Air Rescue HEMS and EMS team interventions concerning newborns.

**Location of call—*n* (%)**		
Urban area		633 (86.83)
Rural area		96 (13.17)
**Type of mission—*n* (%)**		
Emergency response		148 (20.30)
Medical rescue transport		146 (20.03)
Patient transfer		435 (59.67)
**Aircraft—*n* (%)**		
Helicopter		252 (34.57)
Airplane		477 (65.43)
**Time of call—*n* (%)**		
7 AM–6:59 PM		679 (93.14)
7 PM–6:59 AM		50 (6.86)
**Time of year—*n* (%)**		
Spring		188 (25.79)
Summer		209 (28.67)
Fall		170 (23.32)
Winter		162 (22.22)
**Further management—*n* (%)**		
Handover to a ground neonatal ambulance team		467 (64.06)
Handover to a hospital		169 (23.18)
Handover to a ground emergency medical team		60 (8.23)
Patient left in place		18 (2.47)
Death		15 (2.06)
Response time (min)—M (SD) IQR	53.11 (38.31)	1.00–167.00
Time to arrive on scene (min)—M (SD) IQR	42.76 (27.68)	26.00–52.00
Time spent on scene (min)—M (SD) IQR	22.87 (13.74)	14.00–27.00
Transport time (min)—M (SD) IQR	43.45 (26.43)	31.00–48.00
Patient care duration (min)—M (SD) IQR	77.81 (35.57)	60.00–87.00
Time from takeoff to patient handover (min)—M (SD) IQR	134.89 (60.86)	105.00–156.00
Total mission duration (min)—M (SD) IQR	244.37 (163.70)	104.00–370.00
Distance to scene (km)—M (SD) IQR	251.12 (153.90)	85.80–352.60
Distance to hospital (km)—M (SD) IQR	259.30 (125.17)	182.50–332.10
Return flight distance (km)—M (SD) IQR	143.87 (125.47)	20.60–173.30

M—mean; SD—standard deviation; IQR—interquartile range.

**Table 2:** Characteristics of the newborn patients, the most commonly performed emergency medical procedures, and test results.

**Sex—*n* (%)**		
Female		306 (41.98)
Male		423 (58.02)
**ICD-10 diagnosis—*n* (%)**		
Congenital heart defect		229 (31.41)
Congenital vascular malformations		120 (16.46)
Other congenital malformations		143 (19.62)
Respiratory failure		106 (14.54)
Sudden cardiac arrest		32 (4.39)
Other		99 (13.58)
**Medical emergency procedures—*n* (%)**		
Intravenous cannulation		314 (43.07)
Mechanical ventilation		163 (22.36)
Tracheal intubation		139 (19.07)
Gastric intubation		115 (15.78)
Oxygen therapy		111 (15.23)
Sedation		101 (13.85)
**Test results**		
**ECG—*n* (%)**		
Sinus rhythm		661 (90.67)
Supraventricular tachycardia		34 (4.66)
Asystole/PEA		22 (3.02)
Bradycardia/AV block		12 (1.65)
**Pupil size—*n* (%)**		
Normal		692 (94.92)
Dilated		24 (3.29)
Constricted		13 (1.78)
**Pupil reaction to light—*n* (%)**		
Normal		695 (95.34)
Not reactive		34 (4.66)
**Skin coloration—*n* (%)**		
Normal		554 (75.99)
Pale		110 (15.09)
Jaundiced		65 (8.92)
**Skin moisture—*n* (%)**		
Dry		708 (97.12)
Moist		21 (2.88)
**Clinical findings—*n* (%)**		
Apnea		77 (10.56)
Dyspnea		83 (11.39)
Cyanosis		114 (15.64)
GCS *—M (SD) IQR	12.68 (4.47)	14.00–15.00
GCS **—M (SD) IQR	13.03 (4.11)	15.00–15.00
RTS *—M (SD) IQR	6.40 (5.75)	0.00–12.00
RTS **—M (SD) IQR	6.38 (5.72)	0.00–12.00
NACA—M (SD) IQR	3.74 (1.16)	3.00–4.00
Respiration rate *—M (SD) IQR	34.50 (29.07)	25.00–42.00
Respiration rate **—M (SD) IQR	34.15 (16.87)	26.00–42.00
Blood glucose—M (SD) IQR	141.13 (103.14)	84.00–158.00

ICD-10—the International Statistical Classification of Diseases and Related Health Problems; ECG—electrocardiogram; PEA—pulseless electrical activity; AV block—atrioventricular block; GCS—Glasgow Coma Scale; RTS—Revised Trauma Score; NACA—the National Advisory Committee for Aeronautics scale; *—value on arrival; **—value on handover; M—mean; SD—standard deviation; IQR—interquartile range.

**Table 3:** Analysis of correlations between type of mission and selected variable.

Variable	Type of mission			*p*-value
	Emergency response	Medical rescue transport	Patient transfer	
**Location of call—*n* (%)**				
Urban area	58 (39.19)	144 (98.63)	431 (99.08)	0.0000
Rural area	90 (60.81)	2 (1.37)	4 (0.92)	
**Time of call—*n* (%)**				
7 AM–6:59 PM	135 (91.22)	129 (88.36)	415 (95.40)	0.0083
7 PM–6:59 AM	13 (8.78)	17 (11.64)	20 (4.60)	
**ICD-10 diagnosis—*n* (%)**				
Congenital heart defect	2 (1.35)	47 (32.19)	180 (41.38)	0.0000
Congenital vascular malformations	0 (0.00)	40 (27.40)	80 (18.39)	
Other congenital malformations	5 (0.00)	20 (13.70)	118 (27.13)	
Respiratory failure	52 (35.14)	24 (16.44)	30 (6.90)	
Sudden cardiac arrest	28 (18.92)	3 (2.05)	1 (0.23)	
Other	61 (41.22)	12 (8.22)	26 (5.98)	
**Clinical findings—*n* (%)**				
Apnea	26 (17.57)	25 (17.12)	26 (5.98)	0.0000
Dyspnea	14 (9.46)	20 (13.70)	49 (11.26)	0.5156
Cyanosis	46 (31.08)	29 (19.86)	39 (8.97)	0.0000
**Medical emergency procedures—*n* (%)**				
Intravenous cannulation	30 (20.27)	66 (45.21)	218 (50.11)	0.0000
Mechanical ventilation	23 (15.54)	64 (43.84)	76 (17.47)	0.0000
Tracheal intubation	34 (22.97)	55 (37.67)	50 (11.49)	0.0000
Gastric intubation	6 (4.05)	33 (22.60)	76 (17.47)	0.0000
Oxygen therapy	46 (31.08)	19 (13.01)	46 (10.57)	0.0000
Sedation	9 (6.08)	39 (26.71)	53 (12.18)	0.0000
Time to arrive on scene (min)—M (SD)	16.36 (8.01)	50.26 (29.72)	50.50 (25.45)	0.0000
Time spent on scene (min)—M (SD)	25.85 (17.37)	26.71 (15.87)	20.95 (11.56)	0.0001
Transport time (min)—M (SD)	15.46 (6.52)	44.20 (21.54)	49.34 (26.74)	0.0000
Patient care duration (min)—M (SD)	49.40 (28.97)	88.60 (30.92)	83.80 (34.18)	0.0000
Distance to scene (km)—M (SD) IQR	39.47 (21.22)	292.79 (167.93)	319.61 (90.60)	0.0000
Distance to hospital (km)—M (SD) IQR	42.19 (23.58)	243.21 (105.25)	312.29 (85.32)	0.0000

ICD-10—the International Statistical Classification of Diseases and Related Health Problems; M—mean; SD—standard deviation; IQR—interquartile range.

**Table 4:** Incidence of selected health problems among the studied newborns broken down by sex.

ICD-10 Diagnosis	Sex		OR	95% CI	*p*-Value
	Female *	Male			
Congenital heart defect	86 (28.10)	143 (33.81)	0.77	0.56–1.06	0.0322
Congenital vascular malformations	44 (14.38)	76 (17.97)	0.77	0.51–1.15	
Other congenital malformations	56 (18.30)	87 (20.57)	0.87	0.60–1.26	
Respiratory failure	53 (17.32)	53 (12.53)	1.46	0.97–2.21	
Sudden cardiac arrest	19 (6.21)	13 (3.07)	2.09	1.02–4.30	
Other	48 (15.69)	51 (12.06)	1.36	0.89–2.08	

*—reference variable.

## Data Availability

The data presented in this study are available on reasonable request from the corresponding author.
